# Trends in Infectious Disease Mortality, South Korea, 1983–2015

**DOI:** 10.3201/eid2402.170862

**Published:** 2018-02

**Authors:** Young June Choe, Seung-Ah Choe, Sung-Il Cho

**Affiliations:** Brown University, Providence, Rhode Island, USA (Y.J. Choe);; Seoul National University, Seoul, South Korea (Y.J. Choe, S.-I. Cho);; CHA University, Pocheon-si, South Korea (S.-A. Choe)

**Keywords:** South Korea, infectious diseases, deaths, mortality, trend, disparity, inequality, inequity, respiratory infections, sepsis, tuberculosis, vaccine-preventable diseases, inequality

## Abstract

We used national statistics from 1983–2015 to evaluate trends in mortality caused by infectious diseases in South Korea. Age-standardized mortality from infectious disease decreased from 43.5/100,000 population in 1983 to 16.5/100,000 in 1996, and then increased to 44.6/100,000 in 2015. Tuberculosis was the most common cause of death in 1983 and respiratory tract infections in 2015. We observed a significant decline in infant deaths caused by infectious diseases, but mortality in persons age >65 years increased from 135 deaths/100,000 population in 1996 to 307/100,000 in 2015. The relative inequality indices for respiratory tract infections, sepsis, and tuberculosis tended to increase over time. Although substantial progress has been achieved in terms of infant mortality, death rates from infectious disease has not decreased overall. Elderly populations with lower education levels and subgroups susceptible to respiratory infections and sepsis should be the focus of preventive policies.

In many industrialized countries, deaths caused by infectious disease have decreased after introduction of various public health measures, including improvements in hygiene, vaccination, and antimicrobial therapy. However, the epidemiology of infectious diseases is complicated, and such diseases account for a considerable number of deaths globally (*1*). Emergence and reemergence of infectious diseases substantially affect the health outcomes of various populations (*2*). Moreover, deaths caused by infectious diseases have disproportionately affected countries and populations of lower socioeconomic status (*3*).

South Korea has undergone rapid economic growth over the past few decades and over the same period has developed a sustainable healthcare system that is generally accessible (*4*). The overall mortality rate in this country decreased substantially from 1,203–1,665 deaths/100,000 population in 1983 to 587–638/100,000 in 2012; the decrease was associated with rising average life expectancy, from 67 years in 1983 to 81 years in 2012 (*5*,*6*). Despite these changes, persons in South Korea remain vulnerable to infectious diseases that typically impose great burdens on healthcare systems (*7*). Particularly vulnerable populations are disproportionately affected by endemic infections from tuberculosis (TB) and vectorborne pathogens (*8*,*9*). The emergence of pandemic influenza A(H1N1) in 2009 and Middle East respiratory syndrome in 2015 substantially affected not only overall health outcomes but also societal stability (*10*,*11*).

Infectious diseases constitute substantial healthcare burdens even in industrialized countries such as South Korea. The epidemiologic patterns of these diseases change over time. Therefore, exploring changes in population health during phases of economic growth is necessary. Here we describe trends in mortality caused by infectious diseases in South Korea during 1983–2015 and aim to identify factors possibly influencing the observed trends.

## Methods

In South Korea, physicians are legally required to complete death certificates, which are then sent to Statistics Korea and made publicly available through the Korean Statistics Information Service (KOSIS; http://kosis.kr/eng). The death certificate includes information on disease directly leading to death, antecedent causes, and other major conditions contributing to death (*12*). Once the death certificate is submitted, a professional staff in Statistics Korea identifies a single most relevant underlying cause for each death, in accordance with the World Health Organization definition (*13*). The coding is reviewed by a committee to finalize the national public data provided to the users. Nationally, ≈90% of all deaths were certified in 1987, and almost 100% of deaths were certified by 2007 (*14*,*15*). The causes of death are coded according to the Korean Classification of Diseases, Sixth Revision, which is based on the International Classification of Diseases (ICD), 10th Revision (ICD-10). The Korean Classification of Diseases was first adopted in 1973 and was revised in accordance with the amendments in the ICD scheme. The death data collected before 1995, which were coded in the ICD’s Ninth Revision codes, are converted to ICD-10 codes by Statistics Korea by using a mapping reference table (https://kssc.kostat.go.kr:8443).

We retrieved infectious disease mortality rates for 1983–2015 from the KOSIS database and identified the infectious disease groups listed (Table 1). We calculated age-standardized mortality rates for 1983–2015 by using the World Standard Population as reference (*16*,*17*). We analyzed trends by age group (<1, 1–4, 5–14, 15–64, and >65 years) and disease (respiratory tract infection, sepsis, TB, intestinal infection, vaccine-preventable disease, central nervous system [CNS] infection, viral hepatitis, HIV-caused disease, and rheumatic heart disease). We used joinpoint regression analysis to identify years associated with significant changes in mortality rates (*18*). We calculated annual percentage changes (APCs) by using generalized linear models, assuming that a Poisson distribution was in play. To assess the trend over an interval, we computed an average of the APCs from the joinpoint model and used that result as a summary measure. The p value for a 2-sided test for which the true average of the APCs was zero was calculated based on a t distribution (https://surveillance.cancer.gov/help/joinpoint/setting-parameters/method-and-parameters-tab/average-annual-percent-change-aapc).

**Table 1:** Selected infectious disease groups and corresponding ICD-10 codes*

Group	ICD-10 codes
All infectious diseases	A00–B99, J09–J18, J86, G00, G03, G04, I00–I09, M86
Intestinal infections	A00–A09
Tuberculosis	A15–A19
Vaccine-preventable diseases†	A33–A37, B05, B26, B83.0
Sepsis	A40–A41
Viral hepatitis	B15–B19
HIV-related diseases	B20–B24
Central nervous system infections	G00, G03, G04
Rheumatic heart diseases	I00–I09
Respiratory tract infections‡	J09–J18, J86

*ICD-10, International Classification of Diseases, 10th Revision. †Includes diphtheria, tetanus, pertussis, measles, mumps, Japanese encephalitis. ‡Includes influenza, pneumonia, empyema.

We obtained population denominators according to age and education level from the Korean Population and Housing Census data, which are obtained at 5-year intervals. The number of deaths according to age and education level (numerators) calculated from raw death certificate data were available from the KOSIS database. We selected information from the years 2000, 2005, 2010, and 2015 to use as population denominators for calculating mortality rates according to education levels. We analyzed changes in age-standardized mortality associated with selected infectious diseases, by education level, among those age >65 years. We categorized education levels as middle school or less, high school, and college or higher. We calculated the slope index of inequality (SII) and the relative index of inequality (RII) to assess the contributions made by socioeconomic disparity to infectious disease–caused mortality among persons of various education levels (*19*).

We used R Studio version 1.0.136 (R Studio, Boston, MA, USA); the Joinpoint Regression Program version 4.1.0; and the Health Disparities Calculator version 1.2.4 (US National Cancer Institute, Bethesda, MD, USA) in our analyses (*19*,*20*). All personal information was anonymized; the study, therefore, did not require review by our Institutional Ethics Board.

## Results

**Figure 1:**
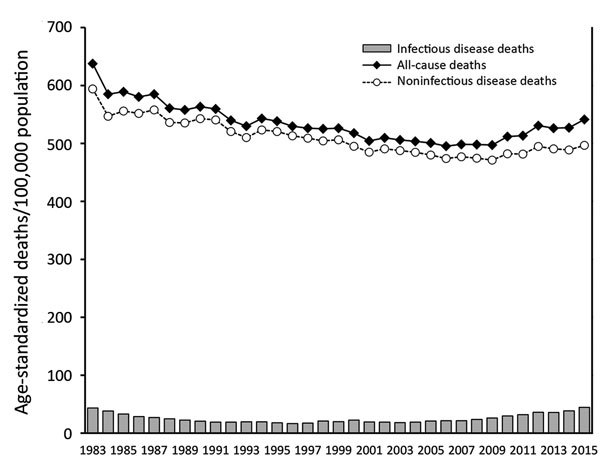
Age-standardized mortality rates (deaths/100,000 population) for infectious diseases, noninfectious diseases, and all causes, South Korea, 1983–2015.

From 1983 through 2015, the number of deaths from infectious diseases exhibited a U-shaped trend, whereas the number of deaths from noninfectious diseases decreased from 594.3 to 496.9/100,000 population (Figure 1). The age-standardized mortality rate from infectious diseases decreased from 43.5/100,000 population in 1983 to 16.5/100,000 in 1996 but then increased to 44.6/100,000 in 2015. In 1983, infectious diseases caused 6.8% (17,376/254,563) of all deaths in South Korea; in 2015, the percentage was 8.3% (22,766/275,895).

TB was the leading cause of infectious disease death in 1983 (7,853 deaths; 23.7/100,000 population) (Table 2). In contrast, in 2015, respiratory tract infections were the most common causes of death from infectious diseases (15,030 deaths; 19.5/100,000 population). Of the top 5 infectious diseases (by number of deaths per 100,000 population), CNS infections and vaccine-preventable diseases (on the 1983 list) were replaced by sepsis and viral hepatitis (on the 2015 list).

**Table 2:** Leading causes of death from infectious disease, South Korea, 1983–2015

Rank	1983				2015		
	Infectious disease	No. deaths	Mortality rate*		Infectious disease	No. deaths	Mortality rate*
1	Tuberculosis	7,853	23.7		Respiratory tract infections	15,030	19.5
2	Respiratory tract infections	5,286	15.1		Sepsis	3,045	4.0
3	Intestinal infections	1,314	4.3		Tuberculosis	2,209	3.0
4	Central nervous system infections	1,244	3.1		Intestinal infections	670	1.0
5	Vaccine-preventable diseases	509	1.1		Viral hepatitis	667	1.0

*Age-standardized mortality rate (deaths/100,000 population).

**Figure 2:**
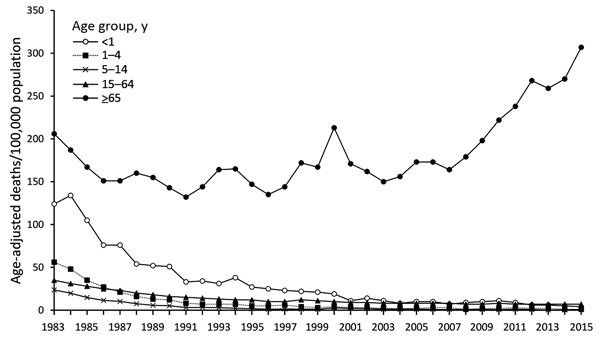
Age-specific infectious disease mortality rates, South Korea, 1983–2015.

We analyzed mortality rates by age group (Figure 2). Infant mortality (i.e., in persons age <1 year) was >100 deaths/100,000 population during 1983–1985, decreased to 38/100,000 in 1994, and fell further to <10/100,000 in 2011. The trends in mortality caused by infectious diseases varied among elderly populations. Among persons age >65 years, mortality rates were 206 deaths/100,000 population in 1983, 135/100,000 in 1996, and 307/100,000 in 2015.

We analyzed trends in age-specific infectious disease mortality rates by using joinpoint analysis (Table 3). Among infants <1 year of age, 3 periods of change were evident. The first period (1983–2007) was associated with an APC of –10.86, the second period (2007–2010) with an APC of 14.56, and the third period (2010–2015) with an APC of –18.45. Overall, the average APC for 1983–2015 was –9.41, reflecting a significant decrease from 124.2 to 4.1 deaths/100,000 population. Among persons age >65 years, the average APC did not vary significantly during this period (APC 1.44), but the APC increased significantly during 2007–2015 (APC 7.70).

**Table 3:** Trends in age-specific mortality rates associated with infectious disease, derived using joinpoint analysis, South Korea, 1983–2015*

Age group, y	Mortality rate		AAPC, 1983–2015	Trend 1			Trend 2			Trend 3	
	1983	2015		Period	APC		Period	APC		Period	APC
<1	124.2	4.1	**−9.41**	1983–2007	**−10.86**		2007–2010	14.56		2010–2015	**−18.45**
1–4	56.3	1.3	**−10.09**	1983–1992	**−21.30**		1992–2015	**−7.02**			
5–14	23.7	0.7	**−8.74**	1983–1996	**−19.70**		1996–2015	−1.72			
15–64	35.3	7.0	**−4.62**	1983–1992	**−10.02**		1992–2005	**−3.99**		2005–2015	−1.36
>65	205.6	307.0	1.44	1983–1986	−10.77		1986–2007	0.72		2007–2015	**7.70**

*AAPC, average annual percentage change; APC, annual percentage change in age-standardized mortality rate. Mortality rates expressed as deaths/100,000 population. Values in bold indicate where AAPC or APC differed significantly (p<0.05) from zero (by a 2-sided test for which the true AAPC was zero, calculated based on a t distribution; https://surveillance.cancer.gov/help/joinpoint/setting-parameters/method-and-parameters-tab/average-annual-percent-change-aapc).

**Figure 3:**
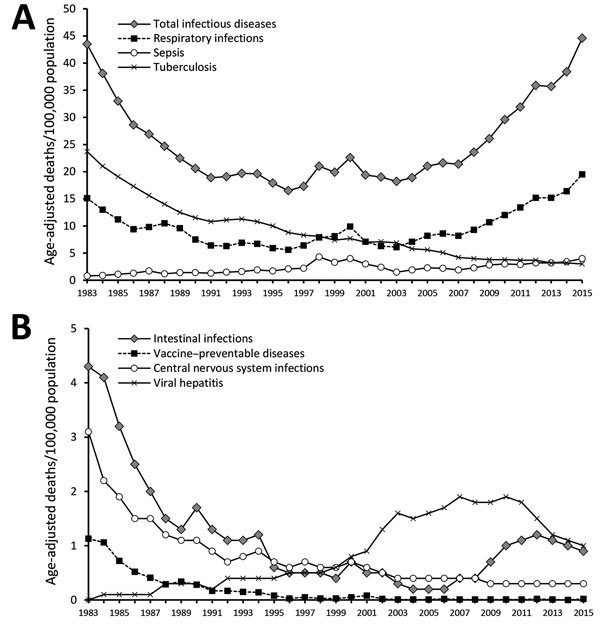
Age-standardized infectious disease mortality rates, South Korea, 1983–2015. A) Mortality rates associated with respiratory infections, sepsis, and tuberculosis. B) Mortality rates associated with intestinal infections, vaccine-preventable diseases, central nervous system infections, and viral hepatitis.

We analyzed age-standardized mortality rates associated with respiratory infections, sepsis, and TB (Figure 3, panel A). TB-related mortality rates decreased during 1983–2015. In 2015, the rate was 3 deaths/100,000 population. During 1983–2015, mortality attributable to respiratory tract infections (pneumonia, empyema, and influenza) increased from 15 to 20 deaths/100,000 population, and sepsis mortality increased from 1 to 4/100,000. We also analyzed age-standardized mortality rates for intestinal infections, vaccine-preventable diseases, CNS infections, and viral hepatitis (Figure 3, panel B). From 1983 to 1999, the mortality rates associated with all of these diseases decreased. Since 2000, the mortality rates associated with intestinal infections and viral hepatitis have gradually increased.

Mortality rates varied by infectious disease; mortality from TB, sepsis, and viral hepatitis exhibited the greatest shifts from 1983 to 2015 (Table 4). The overall average APC (from 1983 to 2015) for respiratory tract infections was 1.06 (15.1 to 19.5 deaths/100,000 population). However, a significant average APC decrease occurred during 1983–1992 (APC −8.89), and a significant increase occurred during 2007–2015 (APC 11.47). The greatest decreases were evident in persons with intestinal infections during 1983–1988 (APC −14.30), in persons with CNS infections during 1983–1986 (APC −13.94), and in persons with vaccine-preventable diseases during 1983–1987 (APC −10.97). The most obvious increases in mortality were in persons with respiratory infections during 2007–2015 (APC 11.47) and sepsis during 2003–2015 (APC 6.99).

**Table 4:** Trends in age-standardized mortality rates associated with infectious disease (derived using joinpoint analysis), South Korea, 1983–2015*

Disease	Mortality rate		AAPC, 1983–2015	Trend 1			Trend 2			Trend 3	
	1983	2015		Period	APC		Period	APC		Period	APC
All infectious disease	43.5	44.6	0.44	1983–1991	**−9.73**		1991–2006	0.67		2006–2015	**8.97**
Respiratory tract infections	15.1	19.5	1.06	1983–1992	**−8.89**		1992–2007	1.83		2007–2015	**11.47**
Sepsis	0.8	4.0	**3.80**	1983–2000	**8.62**		2000–2003	−20.38		2003–2015	**6.99**
Tuberculosis	23.7	3.0	**−5.97**	1983–1989	**−9.12**		1989–2015	**−5.55**			
Intestinal infections	4.3	0.9	**−2.62**	1983–1988	**−14.30**		1988–2004	**−4.50**		2004–2015	**5.48**
Vaccine-preventable diseases	1.1	0.0	**−1.71**	1983–1987	**−10.97**		1987–1996	**−3.00**		1996–2015	**−0.33**
CNS infections	3.1	0.3	**−2.71**	1983–1986	**−13.94**		1986–1992	**−5.50**		1992–2015	**−1.62**
Viral hepatitis	0.0	1.0	**3.24**	1983–1998	**2.75**		1998–2007	**7.92**		2007–2015	**−4.99**
HIV diseases	0.0	0.2	**0.70**	1983–1998	−0.01		1998–2001	2.85		2001–2015	**0.77**
Rheumatic heart diseases	0.5	0.2	−0.35	1983–1999	**−1.40**		1999–2002	9.31		2002–2015	**−2.24**

*AAPC, average annual percentage change; APC, annual percentage change in age-standardized mortality rate; CNS, central nervous system. Mortality rates expressed as deaths/100,000 population. Values in bold indicate where AAPC or APC differed significantly (p<0.05) from zero (by a 2-sided test for which the true AAPC was zero, calculated based on a t distribution; https://surveillance.cancer.gov/help/joinpoint/setting-parameters/method-and-parameters-tab/average-annual-percent-change-aapc).

We stratified mortality rates from infectious diseases by education level for persons >65 years of age (Table 5). For most education levels, the number of deaths attributable to respiratory infections and sepsis tended to decrease during 2000–2010 but increased in 2015. Tuberculosis mortality rates decreased among those of higher education levels during 2000–2015. The SII of respiratory tract infection–associated mortality increased from 46.2 in 2000 to 78.9 in 2015, and the SII of TB-associated mortality fell from 42.0 to 15.2. The respiratory tract infection RII score tended to increase over time. In 2000, the RII was 0.6 but increased to 0.8 in 2005, 1.1 in 2010, and 1.5 in 2015. From 2000 to 2015, the RIIs of sepsis increased from 1.0 to 1.8, and RIIs of TB increased from 0.7 to 1.4.

**Table 5:** Changes in age-standardized mortality rates associated with respiratory tract infections, sepsis, and tuberculosis among adults >65 years of age, by education level and inequality index, South Korea, 2000–2015

Disease by education level and inequality	Mortality rate			
	2000	2005	2010	2015
Respiratory tract infection				
Education level				
Middle school or less	98.6	90.8	60.2	87.9
High school	69.4	53.5	28.6	35.9
College or higher	67.8	55.9	31.1	35.3
Inequality index				
Slope index of inequality	46.2	52.4	43.7	78.9
Relative index of inequality	0.6	0.8	1.1	1.5
Sepsis				
Education level				
Middle school or less	33.9	21.7	14.2	17.3
High school	24.4	12.5	6.1	5.9
College or higher	17.0	10.2	5.2	5.6
Inequality index				
Slope index of inequality	25.4	17.3	13.5	17.6
Relative index of inequality	1.0	1.2	1.6	1.8
Tuberculosis				
Education level				
Middle school or less	60.0	44.3	15.1	13.9
High school	45.8	34.6	7.7	6.1
College or higher	30.1	21.4	5.1	5.9
Inequality index				
Slope index of inequality	42.0	30.7	16.8	15.2
Relative index of inequality	0.7	0.7	1.3	1.4

## Discussion

Despite the reduction in all-cause mortality evident in South Korea from 1983 to 2015, infectious diseases remain problematic. The mortality trend for such diseases is U-shaped; a significant decrease is evident from 1983 to 1991 (APC −9.73), followed by an increase from 2006 to 2015 (APC 8.97). During the period of decrease, the largest percentage change was evident in death rates associated with intestinal infections (APC −14.30%), followed by death rates associated with CNS infections (APC −13.94), vaccine-preventable diseases (APC −10.97), and TB (APC −9.12). South Korea underwent rapid economic development in the 1980s, which was associated with the implementation of accessible universal healthcare in 1989. In 2000, an aging society was evident in South Korea, when the proportion of the population >65 years of age was 7.2% (*6*); this proportion increased to 10.7% in 2010 and to 12.7% in 2014. Improvements in healthcare access and public hygiene might have acted as the key drivers of the decrease in infectious disease mortality (*5*). Surprisingly, however, respiratory infection–associated mortality has increased (APC 11.47), as has mortality attributable to sepsis (APC 6.99) and to intestinal infections (APC 5.48). A major societal turning point occurred in 1997; an economic crisis increased income inequality and weakened social cohesion, which affected health (*21*). The changes in societal structure and the resulting policies have been influenced by efforts to expand flexible labor markets. While workers have been experiencing job insecurity and disadvantages in wages and benefits, the economic crisis has exacerbated health inequalities; recent studies have shown increasing socioeconomic inequalities in self-rated health, suicide, and infant mortality (*21*). The increasing health inequalities are an important aspect of the recent increase in infectious disease–caused mortality in South Korea. Health inequalities are not only an ethics and social justice issue but also an important public health problem that needs to be addressed appropriately. Addressing health inequalities should be prioritized in public health policy for the prevention and control of infectious diseases in South Korea.

We also found that the reductions in infectious disease–related mortality rates noted in the 1980s were largely attributable to fewer infant deaths and TB-related deaths. The greatest declines in infectious disease–related infant mortality were observed during 1983–2007 (APC –10.86) and 2010–2015 (APC −18.45). In South Korea, large corporations were mandated by law to provide health insurance, commencing in 1977, and such coverage was gradually expanded to include the entire population by 1989 (*22*). Improved healthcare access has greatly increased vaccination and antibiotic treatment of infectious diseases. Infants are very susceptible to vaccine-preventable diseases; thus, the introduction of publicly available vaccines and expansion of vaccination coverage was effective in preventing infectious disease deaths. In 1991, the vaccination rates for publicly provided childhood vaccines in South Korea were 56%‒80%, whereas in 2012, the vaccination rates were 95.9%‒100% (*23*,*24*). Moreover, although TB is not uncommon in South Korea, our data provide compelling evidence that deaths from TB have significantly declined over the past 23 years. TB control efforts (improved screening and early treatment) clearly have been successful in mitigating mortality (*25*).

Again, the most notable event during the study period was the 1997 financial crisis in Asia, which had devastating consequences not just on the economy but also the health of undereducated persons (*26*). Many persons were subjected to layoffs, early retirements, and business failures, which affected major health indices such as healthcare use, rates of chronic and acute disease, and all-cause mortality rates, including that of suicide. Given the limited social security available to the elderly population in South Korea, the impact was more severe in this population, especially among persons with less education. The hospitalization rate decreased during the early stage of the economic crisis. During 1995–1998, rates of chronic disease increased by 27.1% and rates of acute disease by 9.5% (*26*). All-cause mortality began to increase ≈1 year after the crisis (*27*). During 1970–2010, overall mortality decreased by 70%–80%; however, only a minimal decline was evident in persons with less education (*28*). We found that education level affected mortality associated with respiratory tract infections and sepsis. Moreover, the elderly population might have acquired chronic diseases that contribute to the increased incidence and severity of infections. Also, the recent decrease in mortality associated with chronic diseases might have resulted in longer periods of exposure to infectious diseases, increasing the risk for death from certain infections in vulnerable populations (*29*).

Influenza and pneumococcal pneumonia are the 2 vaccine-preventable respiratory infections. Influenza vaccine has been included in South Korea’s national immunization program since 1997, and coverage among those >65 years of age was 79.9% during 2004–2005 (*30*). However, during this period, coverage rates varied by sex and lifestyle (*31*). Influenza vaccination campaigns should focus on underrepresented groups, particularly in the elderly population. The extent of pneumococcal polysaccharide vaccination in the elderly was low (0.8%) in 2005 (*32*). After the introduction of a publicly funded program in 2013, vaccination coverage increased from 5.0% to 57.9% over a 20-month period (*33*). Similar to what had been observed in children, the publicly funded program increased vaccination coverage in South Korea. A survey conducted in 2010 showed that state-sponsored vaccination rates in children reached 95.9%–100%, and vaccination rates among the general public were 30.7%–85.4% (*24*). Nationwide vaccination of underprivileged populations might also help counter unmet medical needs in South Korea, especially in the context of vaccine-preventable respiratory infections.

Changes in population structure and the influences of pathogens on disease development might have affected mortality caused by infectious diseases. The numbers of immunocompromised persons are growing; the number of susceptible persons is increasing as a result of the greater extent of procedural and treatment invasiveness, including that associated with chemotherapy and transplantation (*34*,*35*). Nosocomial infections and antimicrobial resistance might increase both the incidence of and death from sepsis, intestinal infections, and pseudomembranous colitis. Few population-level epidemiologic data on sepsis or pseudomembranous colitis are available in South Korea (*36*). We emphasize, therefore, that public health authorities must note that the trends in deaths caused by sepsis have changed (*37*). The hepatitis A outbreak in South Korea during the late 2000s reflects an immunity gap in young adults (*38*). In South Korea, the seroprevalence of hepatitis A has been changing dynamically in adults 20–39 years of age, the population most affected by the outbreak. The increase in viral hepatitis–related deaths in the 2000s suggests that epidemiologic transition cannot be ignored when formulating public health policies.

Our study had several limitations. First, we evaluated only deaths reported on death certificates; these records only partly capture the burden of infectious disease. The need to identify a specific cause of death might encourage miscoding and thus might bias our estimates. However, a previous validation study showed that ≈67% of the deaths from infectious disease were classified accurately (*39*). Second, the KOSIS database does not provide detailed classification of certain disease groups. For example, perinatal infections (P23 and P35‒39) are included among “certain conditions originating in the perinatal period (P00‒96),” heart infections (I30 and I33) are included among “other heart diseases (I26‒51),” and infections of the kidney and urinary tract (N10‒13) are included among “diseases of the genitourinary system: urinary system (N00‒39).” Therefore, deaths caused by these disease groups were not included in this analysis. Third, the practical classification of the diseases in this study might not reflect the specific public health measures for control and prevention. For example, influenza and hepatitis A and B are now vaccine-preventable diseases, but influenza is still classified as a respiratory infection, and hepatitis A and B are still classified as viral hepatitis. As another example, the coding of sepsis is sometimes inconsistent and not standardized explicitly. Because sepsis develops mostly as a consequence of other disease processes, establishing a causal relationship with death is difficult, resulting in certain variations in coding. Depending on the etiology, certain subcategories of sepsis, CNS infections, lower respiratory tract infections, and intestinal infections can be prevented by vaccination.

Despite these limitations, our study illustrates the changes over the last 2 decades in the nature of infectious disease deaths in South Korea, a country that has undergone rapid economic and societal transformations during this period. Although substantial progress has been made in infectious disease prevention and treatment, the burden of infectious diseases has not diminished, principally because of changes in population structure and disproportionate mortality rates among less educated persons. Periodic estimations of disease burden in South Korea are required to appropriately transition epidemiologic public health measures. Our findings will be useful in implementation and evaluation of control and preventive strategies. Sepsis and lower respiratory tract infection deserve attention as serious public health issues in the undereducated elderly population in South Korea. We recommend additional efforts toward managed care in this population, which can prevent avoidable death caused by infectious diseases. Moreover, additional studies to better understand how this type of disparity has affected disease mortality are warranted.

In conclusion, the overall mortality rate from infectious diseases in South Korea remained unchanged during 1983–2015. Monitoring of infectious disease mortality data can help identify subjects requiring disease control and prevention. The trends illustrate the continued vulnerability of South Korea residents to infectious diseases. Elderly persons and persons with respiratory infections and sepsis require particular attention in terms of disease control and prevention.
